# Interrupted inferior vena cava and partial anomalous pulmonary venous return with atrial septal defect in a 38-year-old adult: a case report

**DOI:** 10.4076/1757-1626-2-7346

**Published:** 2009-07-16

**Authors:** Mehmet Cengiz Colak, Ali Rahman, Hasan Kocaturk, Ednan Bayram, Ercan Kocakoc

**Affiliations:** 1 Department of Cardiovascular Surgery, Faculty of Medicine, Firat University23119, ElazigTurkey; 2 Department of Cardiology, Şifa HospitalErzurumTurkey; 3 Department of Radiology, Faculty of Medicine, Firat University23119, ElazigTurkey

## Abstract

We present a woman having congenital anomalies of the inferior vena cava and partial anomalous pulmonary venous return from the right lung with atrial septal defect in a 38-year-old. Congenital anomalies of inferior vena cava are rare. They are seen more often in young males. If there are not other anomalies, they are latent for a long time. Peripheral venous thrombosis, chronic venous insufficiency, dyspnea and fatigue are often the first symptoms of these anomalies. Surgical repair of atrial septal defect with partial anomalous pulmonary venous return include provision of durably unobstructed systemic and pulmonary venous pathways, closure of the atrial septal defect, and avoidance of arrhythmias. The diagnosis has been determined by compression ultrasonography with color doppler assessment, multidetector computed tomography angiography and echocardiography.

## Introduction

Anomalies in the inferior vena cava (IVC) occur in 0.3% of otherwise healthy individuals and in 0.6-2% of patients with other cardiovascular defects [1], but this information could be underestimated, since such anomalies are usually asymptomatic and casually discovered in imaging examinations or abdomen surgery. It is commonly associated with other congenital heart disease [2,3]. Absence of the hepatic segment of the inferior vena cava (IVC), with azygos continuation into the right or left superior vena cava (SVC) has been reported as an incidental finding at postmortem examinations since 1793 [4].

Partial anomalous pulmonary venous connection (PAPVC) is present in approximately 90% of patients with atrial septal defect (ASD) [5]. This represents a physiologic left-to-right shunt with subsequent risk for pulmonary vascular disease, Eisenmenger syndrome, and biventricular failure [6]. Four basic anatomic types of anomalous venous connection have been defined based on the level of the pulmonary systemic (venous) connection relative to the heart and include the following: supracardiac (type I), intracardiac (type II), infracardiac (type III), and mixed drainage (type IV) [5]. Although uncommon, patients without obstruction and without significant heart failure can present later in life and even during adulthood [5].

## Case presentation

**Figure 1.**
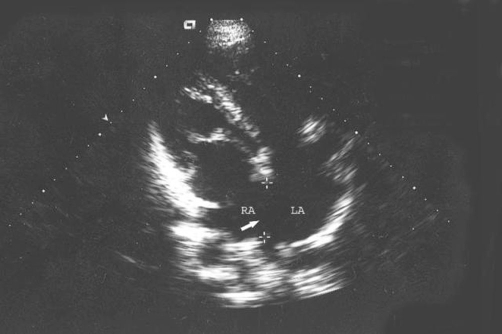
Preoperative echocardiographic evaluation shows atrial septal defect (arrow; between calipers; RA: right atrium, LA: left atrium).

A 38-year-old woman from Elazig, Turkey with atrial septal defect was admitted for exertional dyspnea and fatigue. She had a general tendency and swelling both of the lower limbs at term (38-40 weeks’ gestation). Echocardiography had documented atrial septal defect (Figure 1) and she had three children in alive. She had an intrauterine death at 28 weeks’ gestation.

**Figure 2.**
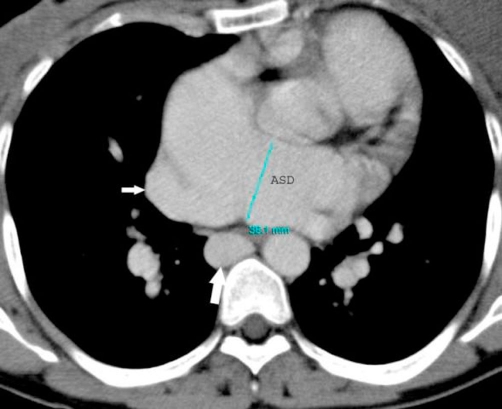
Contrast-enhanced axial CT image demonstrates enlarged azygos (white large arrow), abnormal return of right-sided pulmonary veins into the right atrium (white small arrow) and atrial septal defect (ASD).

**Figure 3.**
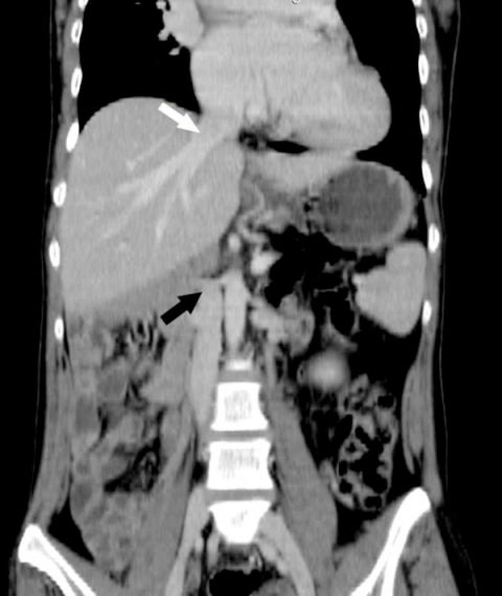
Coronal multiplanar reformation CT image demonstrates the absence of inferior vena cava (black arrow) and hepatic veins draining directly into the right atrium (white arrow).

**Figure 4.**
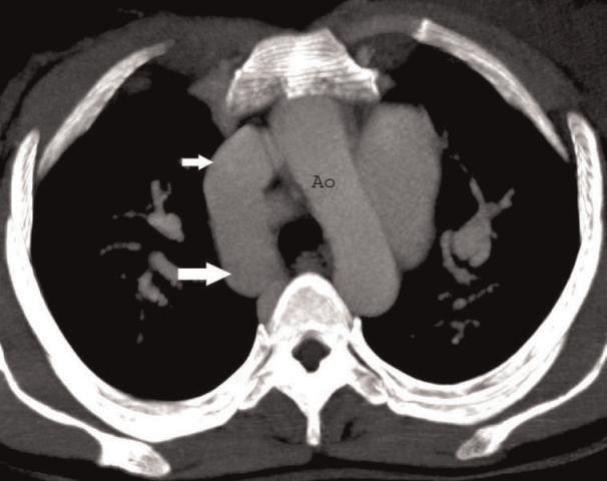
Axial CT image demonstrates vena cava superior (white small arrow) and enlarged azygos vein (white large arrow). (Ao: Aorta).

**Figure 5.**
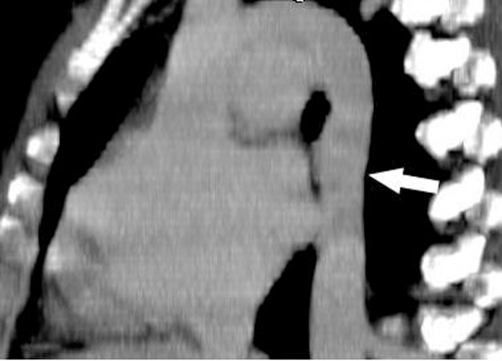
CT axial image that demonstrates enlarged azygos vein, draining into the superior vena cava (Arrow).

**Figure 6.**
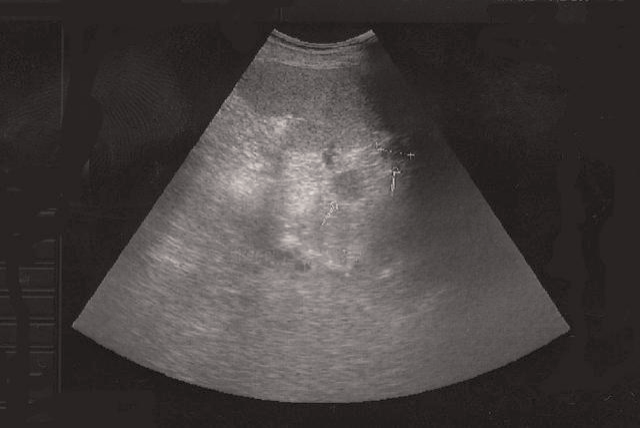
Ultrasound shows two nodular lesions consistent with polysplenia (arrows).

On physical examination, she presented in both lower limbs swelling. A venous color flow Doppler ultrasonography of the both lower-extremity were requested, showing bilateral venous insufficiency, but there were not peripheral venous thrombosis. On auscultation, fixed split of second heart sound and pansystolic murmur on the left side of sternal border were heard. ECG was NSR. A chest X-ray showed mild heart enlargement. Echocardiography documented enlargement of the right ventricle and both atria with atrial septal defect (approximately 33 × 38 mm) and normal function of both ventricles (Figure 1). Right-sided pulmonary veins were not identified. Multidetector Computerized Tomography (CT) angiography confirmed atrial septal defect and abnormal return of right-sided pulmonary veins into the right atrium (Figure 2) and showed inferior vena cava on the right side of spine and showed total interruption of IVC below the hepatic veins (Figure 3). Multidetector CT angiography provided full description of a complex anomaly documenting right inferior vena cava with azygous continuation, draining into the superior vena cava (Figure 4, 5) and hepatic veins were draining directly into the right atrium (Figure 3). Ultrasound revealed polysplenia (Figure 6), 0.5 cm nodule in the left thyroid lobe and 1 cm cystic mass in the right over.

**Figure 7.**
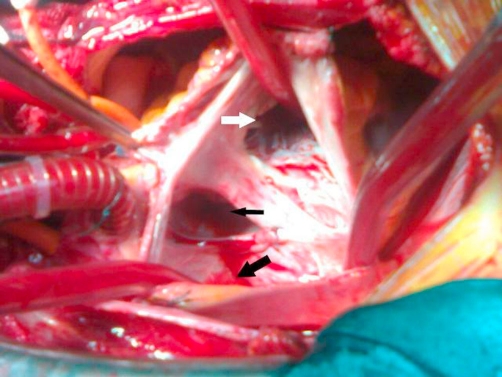
Intraoperative appearance. Tricuspite leaflet (white arrow), atrial septal defect (black small arrow), abnormal return of right-sided pulmonary veins into the right atrium (black large arrow).

**Figure 8.**
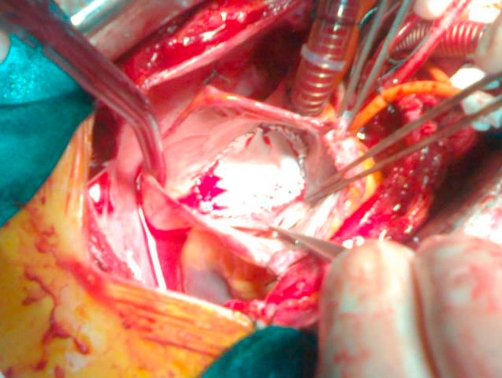
Postop. Repair of the atrial septal defect (ASD) with a patch.

A standard median sternotomy was performed. The pericardial cavity was opened and the heart was exposed. SVC was dissected out to the bridging vein, exposing the azygos vein and anomalous pulmonary veins. Cardiopulmonary bypass was established with cannulation to the ascending aorta and SVC was cannulated by L-shaped catheter, because of absence the IVC. The IVC cannula was not cannulated. After cross-clamping of the ascending aorta, cardiac arrest was obtained using St. Thomas solution through the root cannula under moderate hypothermia. A tourniquet was applied to the SVC and the anomaly was approached by a longitudinal right atriotomy parallel to the atrioventricular sulcus. We saw atrial septal defect and abnormal return of right-sided pulmonary veins into the right atrium (Figure 7). We performed intraatrial rerouting and to drain the anomalous pulmonary veins into the left atrial cavity, using a patch made of a polytetrafluoroethylene (PTFE) in a patient with 5-0 polypropylene (Figure 8). The suture line of the patch was placed away from the sinus node and the pulmonary veins. After the operation sinus rhythm was recovered. There were no complications with follow-up of one year.

## Discussion

In this paper, we present firstly in literature a case with complex congenital anomaly including infrahepatic IVC interruption with azygos continuation and partial pulmonary venous return associated with ASD.

The IVC is composed of four segments: hepatic, suprarenal, renal and infrarenal. All four segments are derived from three embryological venous channels that develop during the 6th to 8th gestational weeks - the postcardinal, subcardinal and supracardinal veins, in order of appearance, respectively. The infrarenal portion of the IVC is believed to arise from the right supracardinal vein. The azygos vein is also derived from the upper segment of the right supracardinal vein, and the hemiazygos is from the upper left supracardinal vein [7,8].

Absence of IVC infrarenal segment is an extremely rare anomaly. Previous studies reported that only 6% of IVC anomalies occur in the renal or infrarenal segments [8], but is more frequently associated with other cardiovascular malformations (dextracardia, septal defects, transposition of the great vessels, pulmonary artery stenosis, common atrium) and situs anomalies, such as left isomerism (polysplenia syndrome) [2].

Infrahepatic IVC interruption with azygos continuation is a rare congenital anomaly, especially when it is not associated with congenital heart disease. Its prevalence is 0.6-2.0% in patients with congenital heart disease and less than 0.3% among otherwise normal patients [9]. This developmental anomaly results in termination of the IVC below the hepatic vein. Systemic venous flow beyond this point is accommodated by the dilated azygos and hemiazygos veins, which eventually empty into the superior vena cava through a dilated azygos arch [10]. In our case was diagnosed partial pulmonary venous return anomalous with ASD in adult and polysplenia and infrahepatic IVC interruption with azygos continuation.

Failure of the hepatic and prerenal segments to fuse is the most common developmental anomaly of the IVC and results in infrahepatic IVC interruption. The infrahepatic IVC may continue as the azygos vein [10,11] or may continue as the hemiazygos vein to the left superior vena cava, intrathoracic veins [12] or anomalous intrahepatic veins [13]. The hepatic segment of the IVC drains directly into the right atrium as in our case [3]. The dilated azygos vein may be misinterpreted as a paracardiac or mediastinal mass on chest radiography [3]. The anomaly may be associated with recurrent deep vein thrombosis of the lower limbs [3], bilateral venous insufficiency as in our case or with sick sinus syndrome [14]. There can be procedural difficulties during right heart catheterisation [15], electrophysiological studies [14], cardiopulmonary bypass surgery, femoral vein catheter advancement, IVC filter placement, and temporary pacing through the transfemoral route [3].

## Conclusion

Combine interrupted inferior vena cava and partial anomalous pulmonary venous returns with ASD are very rare. They can be seen in young males. They can be latent for a long time. Peripheral venous thrombosis, dyspnea and fatigue are often the first symptoms of these anomalies. This case report should emphasize the importance of thorough preoperative evaluation of both systemic and pulmonary venous returns in subjects with congenital heart disease to avoid an injury of abnormally leading large vessels. An IVC abnormality should be excluded. It also illustrates the role of modern imaging techniques in establishing correct diagnosis.

## Abbreviations

ASD: atrial septal defect
CT: Computerized Tomography
IVC: inferior vena cava
PAPVC: Partial anomalous pulmonary venous connection
PTFE: patch made of a polytetrafluoroethylene
SVC: superior vena cava
